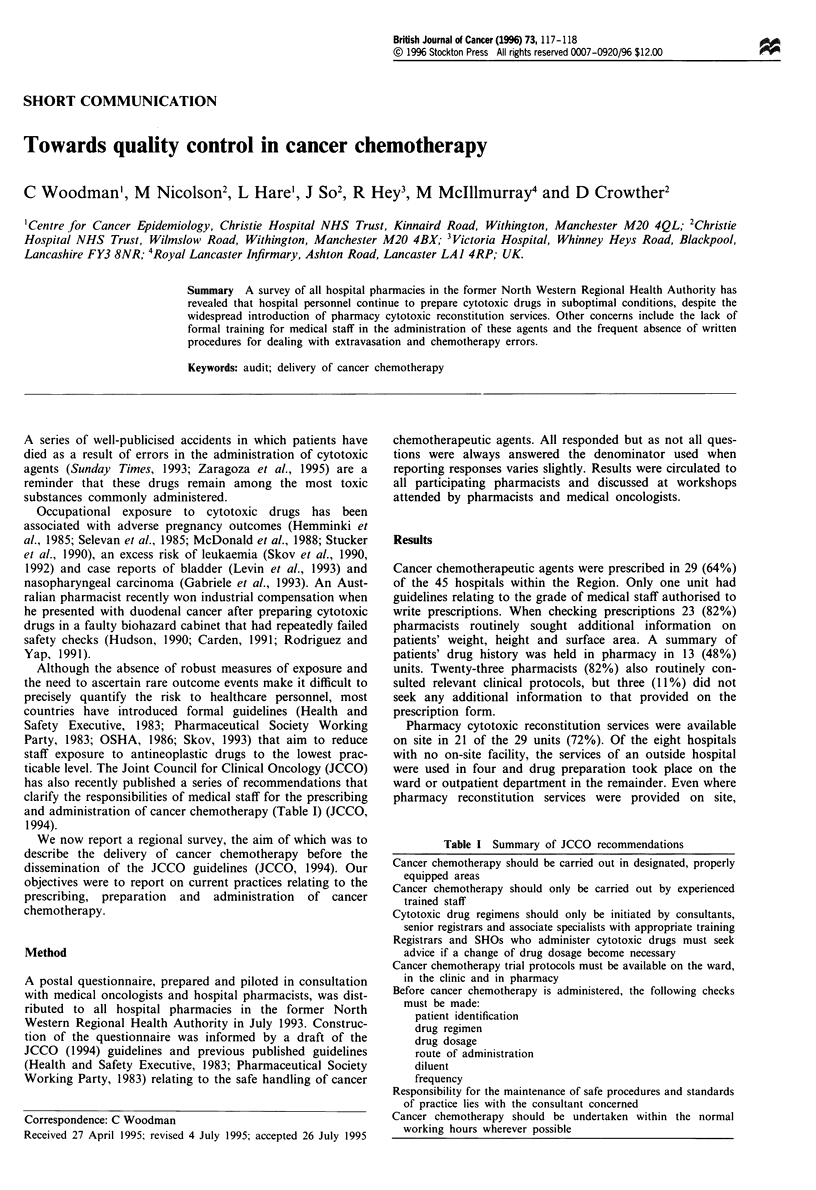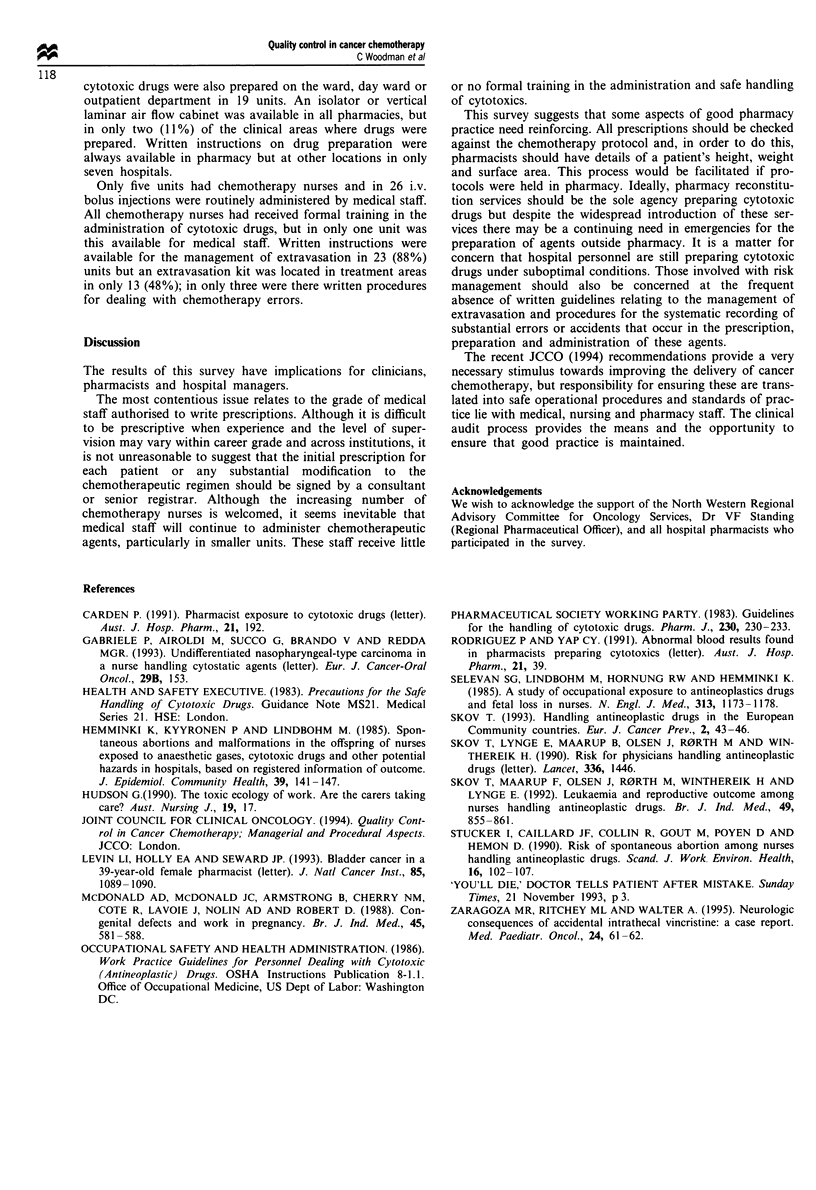# Towards quality control in cancer chemotherapy.

**DOI:** 10.1038/bjc.1996.21

**Published:** 1996-01

**Authors:** C. Woodman, M. Nicolson, L. Hare, J. So, R. Hey, M. McIllmurray, D. Crowther

**Affiliations:** Centre for Cancer Epidemiology, Christie Hospital NHS Trust, Withington, Manchester, UK.

## Abstract

A survey of all hospital pharmacies in the former North Western Regional Health Authority has revealed that hospital personnel continue to prepare cytotoxic drugs in suboptimal conditions, despite the widespread introduction of pharmacy cytotoxic reconstitution services. Other concerns include the lack of formal training for medical staff in the administration of these agents and the frequent absence of written procedures for dealing with extravasation and chemotherapy errors.


British Journal of Cancer (1996) 73, 117-118
© 1996 Stockton Press All rights reserved 0007-0920/96 $12.00

SHORT COMMUNICATION

### Towards quality control in cancer chemotherapy

C Woodman[1], M Nicolson[2], L Hare[1], J So[2], R Hey[3], M McIllmurray[4] and D Crowther[2]

[1]*Centre for Cancer Epidemiology, Christie Hospital NHS Trust, Kinnaird Road, Withington, Manchester M20 4QL;* [2]*Christie Hospital NHS Trust, Wilmslow Road, Withington, Manchester M20 4BX;* [3]*Victoria Hospital, Whinney Heys Road, Blackpool, Lancashire FY3 8NR;* [4]*Royal Lancaster Infirmary, Ashton Road, Lancaster LA1 4RP; UK.*

**Summary** A survey of all hospital pharmacies in the former North Western Regional Health Authority has revealed that hospital personnel continue to prepare cytotoxic drugs in suboptimal conditions, despite the widespread introduction of pharmacy cytotoxic reconstitution services. Other concerns include the lack of formal training for medical staff in the administration of these agents and the frequent absence of written procedures for dealing with extravasation and chemotherapy errors.

**Keywords:** audit; delivery of cancer chemotherapy

A series of well-publicised accidents in which patients have died as a result of errors in the administration of cytotoxic agents (*Sunday Times*, 1993; Zaragoza *et al.*, 1995) are a reminder that these drugs remain among the most toxic substances commonly administered.

Occupational exposure to cytotoxic drugs has been associated with adverse pregnancy outcomes (Hemminki *et al.*, 1985; Selevan *et al.*, 1985; McDonald *et al.*, 1988; Stucker *et al.*, 1990), an excess risk of leukaemia (Skov *et al.*, 1990, 1992) and case reports of bladder (Levin *et al.*, 1993) and nasopharyngeal carcinoma (Gabriele *et al.*, 1993). An Australian pharmacist recently won industrial compensation when he presented with duodenal cancer after preparing cytotoxic drugs in a faulty biohazard cabinet that had repeatedly failed safety checks (Hudson, 1990; Carden, 1991; Rodriguez and Yap, 1991).

Although the absence of robust measures of exposure and the need to ascertain rare outcome events make it difficult to precisely quantify the risk to healthcare personnel, most countries have introduced formal guidelines (Health and Safety Executive, 1983; Pharmaceutical Society Working Party, 1983; OSHA, 1986; Skov, 1993) that aim to reduce staff exposure to antineoplastic drugs to the lowest practicable level. The Joint Council for Clinical Oncology (JCCO) has also recently published a series of recommendations that clarify the responsibilities of medical staff for the prescribing and administration of cancer chemotherapy (Table I) (JCCO, 1994).

We now report a regional survey, the aim of which was to describe the delivery of cancer chemotherapy before the dissemination of the JCCO guidelines (JCCO, 1994). Our objectives were to report on current practices relating to the prescribing, preparation and administration of cancer chemotherapy.

### Method

A postal questionnaire, prepared and piloted in consultation with medical oncologists and hospital pharmacists, was distributed to all hospital pharmacies in the former North Western Regional Health Authority in July 1993. Construction of the questionnaire was informed by a draft of the JCCO (1994) guidelines and previous published guidelines (Health and Safety Executive, 1983; Pharmaceutical Society Working Party, 1983) relating to the safe handling of cancer chemotherapeutic agents. All responded but as not all questions were always answered the denominator used when reporting responses varies slightly. Results were circulated to all participating pharmacists and discussed at workshops attended by pharmacists and medical oncologists.

### Results

Cancer chemotherapeutic agents were prescribed in 29 (64%) of the 45 hospitals within the Region. Only one unit had guidelines relating to the grade of medical staff authorised to write prescriptions. When checking prescriptions 23 (82%) pharmacists routinely sought additional information on patients' weight, height and surface area. A summary of patients' drug history was held in pharmacy in 13 (48%) units. Twenty-three pharmacists (82%) also routinely consulted relevant clinical protocols, but three (11%) did not seek any additional information to that provided on the prescription form.

Pharmacy cytotoxic reconstitution services were available on site in 21 of the 29 units (72%). Of the eight hospitals with no on-site facility, the services of an outside hospital were used in four and drug preparation took place on the ward or outpatient department in the remainder. Even where pharmacy reconstitution services were provided on site,

**Table I** Summary of JCCO recommendations

| |
|---|
| Cancer chemotherapy should be carried out in designated, properly equipped areas |
| Cancer chemotherapy should only be carried out by experienced trained staff |
| Cytotoxic drug regimens should only be initiated by consultants, senior registrars and associate specialists with appropriate training |
| Registrars and SHOs who administer cytotoxic drugs must seek advice if a change of drug dosage become necessary |
| Cancer chemotherapy trial protocols must be available on the ward, in the clinic and in pharmacy |
| Before cancer chemotherapy is administered, the following checks must be made: |
| patient identification |
| drug regimen |
| drug dosage |
| route of administration |
| diluent |
| frequency |
| Responsibility for the maintenance of safe procedures and standards of practice lies with the consultant concerned |
| Cancer chemotherapy should be undertaken within the normal working hours wherever possible |

Correspondence: C Woodman
Received 27 April 1995; revised 4 July 1995; accepted 26 July 1995

cytotoxic drugs were also prepared on the ward, day ward or outpatient department in 19 units. An isolator or vertical laminar air flow cabinet was available in all pharmacies, but in only two (11%) of the clinical areas where drugs were prepared. Written instructions on drug preparation were always available in pharmacy but at other locations in only seven hospitals.

Only five units had chemotherapy nurses and in 26 i.v. bolus injections were routinely administered by medical staff. All chemotherapy nurses had received formal training in the administration of cytotoxic drugs, but in only one unit was this available for medical staff. Written instructions were available for the management of extravasation in 23 (88%) units but an extravasation kit was located in treatment areas in only 13 (48%); in only three were there written procedures for dealing with chemotherapy errors.

### Discussion

The results of this survey have implications for clinicians, pharmacists and hospital managers.

The most contentious issue relates to the grade of medical staff authorised to write prescriptions. Although it is difficult to be prescriptive when experience and the level of supervision may vary within career grade and across institutions, it is not unreasonable to suggest that the initial prescription for each patient or any substantial modification to the chemotherapeutic regimen should be signed by a consultant or senior registrar. Although the increasing number of chemotherapy nurses is welcomed, it seems inevitable that medical staff will continue to administer chemotherapeutic agents, particularly in smaller units. These staff receive little or no formal training in the administration and safe handling of cytotoxics.

This survey suggests that some aspects of good pharmacy practice need reinforcing. All prescriptions should be checked against the chemotherapy protocol and, in order to do this, pharmacists should have details of a patient's height, weight and surface area. This process would be facilitated if protocols were held in pharmacy. Ideally, pharmacy reconstitution services should be the sole agency preparing cytotoxic drugs but despite the widespread introduction of these services there may be a continuing need in emergencies for the preparation of agents outside pharmacy. It is a matter for concern that hospital personnel are still preparing cytotoxic drugs under suboptimal conditions. Those involved with risk management should also be concerned at the frequent absence of written guidelines relating to the management of extravasation and procedures for the systematic recording of substantial errors or accidents that occur in the prescription, preparation and administration of these agents.

The recent JCCO (1994) recommendations provide a very necessary stimulus towards improving the delivery of cancer chemotherapy, but responsibility for ensuring these are translated into safe operational procedures and standards of practice lie with medical, nursing and pharmacy staff. The clinical audit process provides the means and the opportunity to ensure that good practice is maintained.

**Acknowledgements**
We wish to acknowledge the support of the North Western Regional Advisory Committee for Oncology Services, Dr VF Standing (Regional Pharmaceutical Officer), and all hospital pharmacists who participated in the survey.

### References

CARDEN P. (1991). Pharmacist exposure to cytotoxic drugs (letter). *Aust. J. Hosp. Pharm.*, **21**, 192.

GABRIELE P, AIROLDI M, SUCCO G, BRANDO V AND REDDA MGR. (1993). Undifferentiated nasopharyngeal-type carcinoma in a nurse handling cytostatic agents (letter). *Eur. J. Cancer-Oral Oncol.*, **29B**, 153.

HEALTH AND SAFETY EXECUTIVE. (1983). *Precautions for the Safe Handling of Cytotoxic Drugs*. Guidance Note MS21. Medical Series 21. HSE: London.

HEMMINKI K, KYYRONEN P AND LINDBOHM M. (1985). Spontaneous abortions and malformations in the offspring of nurses exposed to anaesthetic gases, cytotoxic drugs and other potential hazards in hospitals, based on registered information of outcome. *J. Epidemiol. Community Health*, **39**, 141-147.

HUDSON G.(1990). The toxic ecology of work. Are the carers taking care? *Aust. Nursing J.*, **19**, 17.

JOINT COUNCIL FOR CLINICAL ONCOLOGY. (1994). *Quality Control in Cancer Chemotherapy; Managerial and Procedural Aspects*. JCCO: London.

LEVIN LI, HOLLY EA AND SEWARD JP. (1993). Bladder cancer in a 39-year-old female pharmacist (letter). *J. Natl Cancer Inst.*, **85**, 1089-1090.

MCDONALD AD, MCDONALD JC, ARMSTRONG B, CHERRY NM, COTE R, LAVOIE J, NOLIN AD AND ROBERT D. (1988). Congenital defects and work in pregnancy. *Br. J. Ind. Med.*, **45**, 581-588.

OCCUPATIONAL SAFETY AND HEALTH ADMINISTRATION. (1986). *Work Practice Guidelines for Personnel Dealing with Cytotoxic (Antineoplastic) Drugs*. OSHA Instructions Publication 8-1.1. Office of Occupational Medicine, US Dept of Labor: Washington DC.

PHARMACEUTICAL SOCIETY WORKING PARTY. (1983). Guidelines for the handling of cytotoxic drugs. *Pharm. J.*, **230**, 230-233.

RODRIGUEZ P AND YAP CY. (1991). Abnormal blood results found in pharmacists preparing cytotoxics (letter). *Aust. J. Hosp. Pharm.*, **21**, 39.

SELEVAN SG, LINDBOHM M, HORNUNG RW AND HEMMINKI K. (1985). A study of occupational exposure to antineoplastics drugs and fetal loss in nurses. *N. Engl. J. Med.*, **313**, 1173-1178.

SKOV T. (1993). Handling antineoplastic drugs in the European Community countries. *Eur. J. Cancer Prev.*, **2**, 43-46.

SKOV T, LYNGE E, MAARUP B, OLSEN J, RØRTH M AND WINTHEREIK H. (1990). Risk for physicians handling antineoplastic drugs (letter). *Lancet*, **336**, 1446.

SKOV T, MAARUP F, OLSEN J, RØRTH M, WINTHEREIK H AND LYNGE E. (1992). Leukaemia and reproductive outcome among nurses handling antineoplastic drugs. *Br. J. Ind. Med.*, **49**, 855-861.

STUCKER I, CAILLARD JF, COLLIN R, GOUT M, POYEN D AND HEMON D. (1990). Risk of spontaneous abortion among nurses handling antineoplastic drugs. *Scand. J. Work. Environ. Health*, **16**, 102-107.

'YOU'LL DIE,' DOCTOR TELLS PATIENT AFTER MISTAKE. *Sunday Times*, 21 November 1993, p 3.

ZARAGOZA MR, RITCHEY ML AND WALTER A. (1995). Neurologic consequences of accidental intrathecal vincristine: a case report. *Med. Paediatr. Oncol.*, **24**, 61-62.